# Resistance of Gram-Negative Bacteria to Eravacycline: A Systematic Review of Data from In Vitro Studies

**DOI:** 10.3390/pathogens14121214

**Published:** 2025-11-28

**Authors:** Matthew E. Falagas, Laura T. Romanos, Dimitrios S. Kontogiannis, Charalampos Filippou, Drosos E. Karageorgopoulos

**Affiliations:** 1 Alfa Institute of Biomedical Sciences (AIBS), 151 23 Marousi, Athens, Greece; l.romanos@aibs.gr (L.T.R.); d.kontogiannis@aibs.gr (D.S.K.); 2 School of Medicine, European University Cyprus, 2404 Nicosia, Cyprus; c.filippou@euc.ac.cy; 3 Department of Medicine, Tufts University School of Medicine, Boston, MA 02111, USA; 4 Department of Infectious Diseases, Oxford University Hospitals NHS Foundation Trust, Oxford OX3 9DU, UK; drkarag@gmail.com

**Keywords:** *E. coli*, *K. pneumoniae*, *E. cloacae*, *A. baumannii*, *P. aeruginosa*, *S. maltophilia*, eravacycline, tetracyclines, fluorocyclines, complicated intra-abdominal infections

## Abstract

Introduction: Eravacycline is a new fluorocycline antibiotic with a broad spectrum of antimicrobial activity approved for the treatment of patients with complicated intra-abdominal infections. This systematic review aimed to evaluate the published data on the resistance of Gram-negative bacterial isolates to eravacycline. Methods: We identified relevant publications by systematically searching Embase, PubMed, Scopus, and Web of Science from their inception to 29 August 2025. Published antimicrobial resistance breakpoints of the European Committee on Antimicrobial Susceptibility Testing (EUCAST) and the US Food and Drug Administration (FDA) were used. Results: Data on 59,922 Gram-negative bacterial clinical isolates were retrieved from 68 articles after the screening of 283 potentially relevant studies. The resistance of consecutive (non-selected) *Escherichia coli* ranged from 0.9% to 9.6%. The MIC_50_ values of eravacycline were ≤0.5 mg/L for *Acinetobacter baumannii* isolates, including carbapenem-resistant *A. baumannii*, in the majority of studies. The proportions of resistance were higher among other lactose non-fermenting Gram-negative bacterial isolates, especially *Pseudomonas aeruginosa,* as well as among selected *E. coli* with advanced patterns of antimicrobial resistance. Conclusions: The evaluated data support the adequate antimicrobial activity of eravacycline against most Gram-negative bacterial clinical isolates. However, in vitro antimicrobial susceptibility testing and modern molecular diagnostic tests, including those that examine mechanisms of resistance, are helpful for the appropriate use of eravacycline in clinical practice.

## 1. Introduction

Tetracyclines are a group of antibiotics, more specifically protein synthesis inhibitors, that have been used in clinical practice for many decades. They act by inhibiting the 30S ribosomal subunit, thereby preventing aminoacyl-tRNA from binding to the acceptor site of the ribosomal complex on the mRNA [1]. Their antimicrobial spectrum is broad, and they have been used by clinicians to treat patients with various types of infections for decades.

Eravacycline, formerly TP-434, is a new synthetic fluorocycline that belongs to the expanded family of tetracycline-type antibiotics [2,3]. It was developed to combat the growing issue of bacterial resistance to tetracyclines. It has a structure similar to that of tigecycline, a glycylcycline antibiotic, with two modifications to its tetracycline core [4]. These modifications were made to bypass common bacterial resistance mechanisms, such as efflux pumps [5].

Its spectrum is broad, covering several Gram-negative, Gram-positive, and anaerobic bacteria, as well as some atypical bacteria [2]. It has shown effectiveness against several species of Enterobacterales and lactose non-fermenting Gram-negative bacteria. *Pseudomonas aeruginosa* is an exception, as it has demonstrated high resistance to this drug [3]. The European Medicines Agency (EMA) and the Food and Drug Administration (FDA) approved it for use in 2018 for the treatment of complicated intra-abdominal infections (cIAI) in adult patients [4,5]. As the nature of these infections is commonly polymicrobial and caused by Gram-negative aerobic bacteria, Gram-positive aerobic bacteria, and anaerobic bacteria, eravacycline is a promising therapeutic option, including other types of infections, particularly those caused by multiresistant bacteria [6].

In this context, we sought to gather available data on the resistance of Gram-negative bacteria to this antimicrobial agent to evaluate its effectiveness against these pathogens. Our article aims to help clinicians understand how they can effectively incorporate this drug into their practice.

## 2. Methods

### 2.1. Sources and Eligibility Criteria

This systematic review was performed in accordance with the Preferred Reporting Items for Systematic Reviews and Meta-Analyses (PRISMA) guidelines. The study protocol was not uploaded to a registry. We performed an extensive literature review across four databases, specifically Embase, PubMed, Scopus, and Web of Science, from their inception to 29 August 2025. Eligible for assessment were studies of any primary research design that met the following inclusion criteria: (a) the terms “eravacycline” or “TP-434” included in the title/abstract/keywords, and (b) the study reported minimum inhibitory concentration (MIC) or disk diffusion zone diameters susceptibility data.

The exclusion criteria were (in the order they were applied): (a) non-primary research articles; (b) articles using isolates from animal sources; (c) case reports of a single isolate or patient; (d) primary research articles that did not contain relevant data for this review; (e) conference abstracts; (f) studies evaluating <10 total isolates, for the eravacycline susceptibility testing, and (g) studies that used only disk diffusion method for in vitro susceptibility testing without use of the broth microdilution method.

### 2.2. Search Strategy

The detailed search strategy is presented in Supplementary File S1. Terms such as “eravacycline”, “resistance”, “non-susceptibility”, “Enterobacteriaceae”, “Enterobacterales”, “*Pseudomonas*”, “*Acinetobacter*”, “*Stenotrophomonas*”, “MIC”, and “disk diffusion” were used.

### 2.3. Screening of Studies

Using the Rayyan tool’s deduplication feature, we deduplicated studies across different databases using their digital object identifiers (DOIs). Two independent reviewers (L.T.R. and D.S.K.) screened all the retrieved records using the full text.

### 2.4. Breakpoints of Susceptibility Testing

At the time of this writing, only limited susceptibility breakpoints had been reported by the relevant committees. For Gram-negative bacteria, the European Committee on Antimicrobial Susceptibility Testing (EUCAST) has published the breakpoints for *Escherichia coli* specifically (susceptible at MIC ≤ 0.5 mg/L). While the Clinical and Laboratory Standards Institute (CLSI) has not yet established breakpoints for this antibiotic, the FDA has set the breakpoint of susceptibility for all Enterobacterales at MIC ≤ 0.5 mg/L.

### 2.5. Data Extraction and Tabulation

The data were grouped by bacterial species (Enterobacterales vs. lactose non-fermenting Gram-negative bacteria). Additionally, we separated the data into consecutive (non-selected) and non-consecutive (selected) isolates, as reported by the study authors. We included data on the total number of isolates studied, the number of isolates of specific species, and the presence of various β-lactamase genes (determined by phenotypic and/or genotypic methods). We also included data on the MIC range, MIC_50_, MIC_90_, and the percentage of resistant isolates. Whenever percentages were provided as the only measure of eravacycline resistance in a study, we calculated the corresponding number of resistant isolates based on the total number of isolates and the given percentage and vice versa. Any disagreements between reviewers were resolved by consensus with a senior author (M.E.F.)

## 3. Results

### 3.1. Selection of Relevant Articles

**Figure 1:**
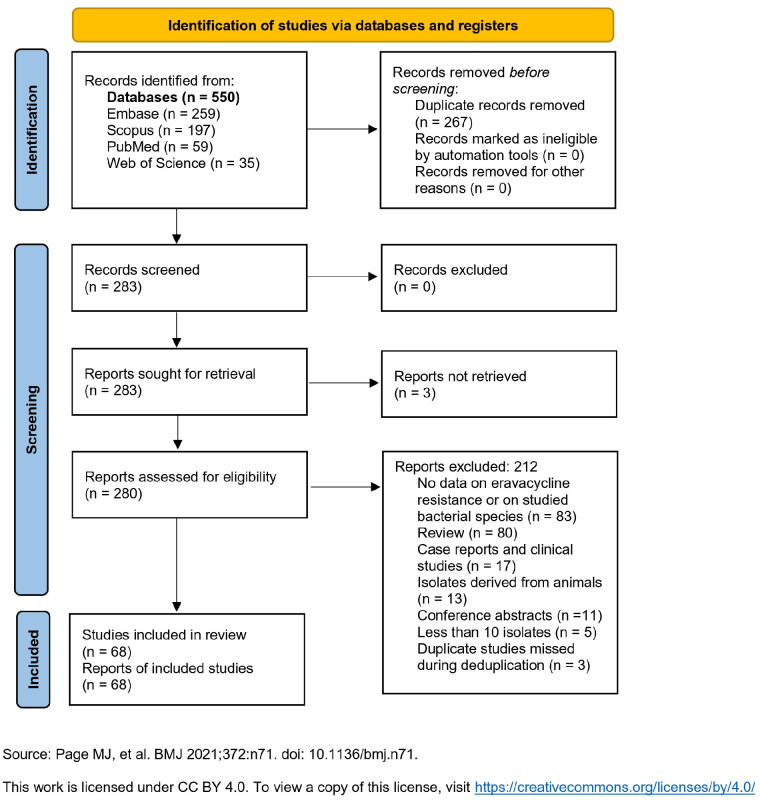
PRISMA 2020 flow diagram for new systematic reviews which included searches of databases and registers only [75].

Figure 1 presents the “Preferred Reporting Items for Systematic Reviews and Meta-Analyses” (PRISMA) flow diagram for evaluating, selecting, and including relevant articles. In total, 550 articles were identified and, after deduplication, 283 were screened. A full-text evaluation was conducted for all 283 articles; after excluding 212, 68 were eligible for inclusion (3 articles could not be retrieved) [7,8,9,10,11,12,13,14,15,16,17,18,19,20,21,22,23,24,25,26,27,28,29,30,31,32,33,34,35,36,37,38,39,40,41,42,43,44,45,46,47,48,49,50,51,52,53,54,55,56,57,58,59,60,61,62,63,64,65,66,67,68,69,70,71,72,73,74].

### 3.2. Main Findings

The evaluated studies included data on 59,922 clinical isolates and were categorized into four groups: studies assessing resistance among (a) consecutive (non-selected) Enterobacterales isolates, (b) selected Enterobacterales isolates, (c) consecutive (non-selected) lactose non-fermenting Gram-negative isolates, and (d) selected lactose non-fermenting Gram-negative isolates.

### 3.3. Resistance of Consecutive Enterobacterales Clinical Isolates to Eravacycline

Table 1 presents data on consecutive (non-selected) Enterobacterales isolates [7,8,9,10,11,12,13,14]. Breakpoints definitions of resistance used by authors varied, with some using EUCAST breakpoints, some FDA breakpoints, and some defining their own breakpoints, based, for example, on the epidemiological cut-off values (ECOFF). Resistance percentages were 0.9% and 9.6% in the two studies that used breakpoints defined by EUCAST [9,11]. These figures involved *E. coli* isolates, as EUCAST breakpoints are only applicable for this species. The isolates in the three studies, which used FDA breakpoints, showed resistance of Enterobacterales to eravacycline ranging from 0.9% to 41.9% [9,10,11]. Specifically, among *E. coli* isolates, the resistance percentages were 0.9% and 9.6% in the two studies with relevant data, as EUCAST and FDA have adopted the same resistance breakpoint for *E. coli* [9,11]. In all five studies that presented specific relevant data for cumulatively 4671 *E. coli* isolates, the eravacycline MIC_90_ was 0.5 mg/L [9,11,12,13,14].

Among *Klebsiella pneumoniae* isolates, resistance was 10% and 41.9% in the two studies with relevant data [9,11]. Among six studies that presented specific, applicable data and included a cumulative total of 2133 *K. pneumoniae* isolates, the MIC_90_ of eravacycline was 0.5 mg/L in one study [11] and >0.5 mg/L in the remaining five studies [7,9,12,13,14]. The MIC_50_ was ≤0.5 mg/L in all six studies.

The resistance of *Enterobacter cloacae* was 18.3% [11]. The MIC_90_ of eravacycline was >0.5 mg/L in all three studies that reported specific, relevant data, totaling 569 *E. cloacae* complex isolates [11,12,14]. The MIC_50_ was 0.5 mg/L in all three studies.

Additionally, in one study, Enterobacterales showed 16.7% resistance when the MIC > 0.5 resistance breakpoint was used, as defined by the authors [8]. In another study, the resistance to eravacycline of all the Enterobacterales species isolates evaluated was 14.3% [10]. The MIC_90_ was 1 mg/L in both studies, which included a total of 1424 isolates [8,10].

### 3.4. Resistance of Selected Enterobacterales Clinical Isolates to Eravacycline

Table 2 presents data on selected Enterobacterales isolates [15,16,17,18,19,20,21,22,23,24,25,26,27,28,29,30,31,32,33,34,35,36,37,38,39,40,41,42,43,44,45,46,47]. Breakpoint variability was also present in these studies, as previously mentioned. Resistance to eravacycline was 0% and 37.5% among *E. coli* isolates in the four studies that used the EUCAST breakpoints [15,16,18,20,28,29,31,33,35,39,40,43]. The authors of three other studies applied the EUCAST breakpoints for *E. coli* to the total number of Enterobacterales isolates. In these three studies, resistance ranged from 0% to 94.1% [20,28,43]. In studies that utilized the FDA breakpoints, resistance of Enterobacterales ranged from 0% to 100% [15,16,17,18,19,22,23,25,26,27,28,29,30,31,32,33,34,35,36,37,38,39,40,41,42,43,44,46,47]. Specifically, in *E. coli* isolates, resistance to eravacycline ranged from 0% to 29% [15,16,17,18,20,26,28,31,33,35,37,38,39,40,42,43,46]. In *K. pneumoniae,* resistance ranged from 0% to 100% [15,17,18,20,26,28,31,33,35,36,37,38,40,42,43,46]. In *E. cloacae/E. cloacae* complex, resistance ranged from 10.5% to 55.6% [18,20,26,28,31,33,40]. In one study in which the authors used MIC ≥ 1 mg/L as the breakpoint for resistance, 23.7% of the total Enterobacterales isolates were resistant. In another study, no resistant isolates were found when the authors used a MIC > 2 mg/L breakpoint for resistance.

### 3.5. Resistance of Consecutive Lactose Non-Fermenting Gram-Negative Bacterial Clinical Isolates to Eravacycline

Table 3 presents data on consecutive lactose non-fermenting isolates [8,9,11,12,13,14,15,28,43,46,48,49,50,51,52,53,54,55,56,57,58]. As there are no available resistance breakpoints for these bacteria, resistance data are reported according to the breakpoints defined by the authors of each article. In one article, where the authors used a resistance breakpoint of MIC > 0.25 mg/L, *Acinetobacter baumannii* isolates showed 43.4% resistance. In the same article, the authors also used a MIC breakpoint of >0.5 mg/L for the same isolates, and the resistance was 28.3% [8]. In another study, authors used the breakpoints for Enterobacterales as defined by EUCAST (MIC > 0.5 mg/L), and 38.6% of isolates were resistant to eravacycline [52].

### 3.6. Resistance of Selected Lactose Non-Fermenting Gram-Negative Bacterial Clinical Isolates to Eravacycline

Table 4 presents data on selected lactose non-fermenting isolates [18,24,26,35,38,44,59,60,61,62,63,64,66,67,68,69,70,71,72,73,74,77]. Resistance breakpoints for these isolates were also defined by some authors. In one study, in which the authors used resistance breakpoints of MIC ≥ 8 mg/L, the resistance was 1.9% [59]. In another study, carbapenem-resistant *A. baumannii* isolates (CRAB) and carbapenem-susceptible *A. baumannii* (CSAB) isolates had resistance percentages of 2.4% and 1.3%, respectively, when the breakpoint for resistance was MIC > 4 mg/L [61]. In one study, a MIC breakpoint of >4 mg/L was used, and *A. baumannii* isolates showed 52.4% resistance [62]. One author used an intermediate resistance breakpoint of MIC 4 mg/L and a resistance breakpoint of MIC ≥ 8 mg/L. Intermediate resistance percentages for both CRAB and CSAB isolates were 1.4%, while resistance percentages were 2% and 0.7%, respectively [63]. In three studies in which the great majority, if not all, of the isolates were carbapenem-resistant, the authors used breakpoints from EUCAST or FDA, or those defined by other authors, which deemed all isolates with MIC > 0.5 mg/L as resistant. The resistance percentages for these studies were 8.9%, 24.1%, and 80.9% [26,38,64].

### 3.7. Eravacycline MIC Distribution for Clinically Important Gram-Negative Bacteria

Among 21 studies that included specific relevant data (Table 1 and Table 2), the MIC_90_ of *E. coli* isolates to eravacycline was 0.5 mg/L or less in 16 studies [9,11,12,13,14,15,16,17,18,28,31,37,39,42,43,46], which included, cumulatively, 10,116 isolates. The MIC_90_ was 1 mg/L in four studies with cumulatively 376 selected isolates [20,35,38,40], and more than 1 mg/L in a single study with eight *E. coli* isolates [33]. The MIC_50_ was less than or equal to 0.5 mg/L in all 21 studies. More specifically, the MIC_50_ was 0.12 mg/L in two studies [12,13], 0.25 mg/L in five studies [9,13,14,35,40], and 0.5 mg/L in three studies (all with selected isolates) [20,33,38].

Among 29 studies that provided data for *K. pneumoniae* or *Klebsiella* spp. isolates, the MIC_90_ of eravacycline was 0.5 mg/L in four studies [11,30,34,43] with cumulatively 5430 isolates. The MIC_90_ was above 0.5 mg/L in the remaining 25 studies, which included a total of 6773 isolates [7,9,12,13,14,15,17,18,19,20,21,23,24,25,28,29,31,33,35,36,38,39,40,41,46]. The MIC_50_ was lower than or equal to 0.5 mg/L in all except for nine studies. The latter nine studies included a total of 945 isolates [17,18,19,29,33,35,38,39,41].

Regarding *Enterobacter cloacae* complex, MIC_50_ and MIC_90_ data were provided in 10 studies, totaling 2746 isolates [11,12,14,18,20,28,30,31,33,40]. The MIC_90_ of eravacycline was 0.5 mg/L or lower in one of these studies [30]. The MIC_50_ was 0.5 mg/L or lower in all but two of these studies [18,31]; the latter included 96 isolates.

Regarding non-fermenting Gram-negative bacteria, specifically *Acinetobacter baumannii* complex, MIC_50_ and MIC_90_ data were provided in 26 studies with, cumulatively, 8744 isolates [9,11,12,13,14,15,18,24,28,35,43,46,48,49,52,55,56,59,60,62,64,66,67,70,73,74]. The MIC_90_ was 0.5 mg/L in four studies [11,13,15,66], which included a total of 682 isolates. The MIC_50_ was 0.5 mg/L or lower in 21 of these studies [9,11,12,13,14,15,18,24,28,43,46,49,52,59,60,64,66,73,74], which included a total of 7832 isolates.

Seventeen studies reported MIC distributions for CRAB or carbapenemase-producing *A. baumannii* complex isolates [8,24,26,46,55,57,58,59,60,61,62,63,64,66,68,73,74]. These studies included 3231 isolates in total. The MIC_90_ was 0.5 mg/L or less in two studies [58,66], with 239 isolates. The MIC_50_ was 0.5 mg/L or less in 10 studies with 2378 isolates [24,46,58,59,60,61,63,66,73,74].

## 4. Discussion

### 4.1. Interpretation of Results

The data analyzed show that the resistance of Enterobacterales isolates to eravacycline varies by species. The drug appears to be effective against *E. coli* isolates (with resistance that ranged from 0.9% to 9.6% among consecutive isolates and from 0% to 29% among selected isolates). However, higher proportions of resistance to eravacycline are noted among isolates of other Enterobacterales species, specifically *K. pneumoniae* and *E. cloacae* complex. Additionally, the proportion of resistance to eravacycline among *Morganellaceae*, including *Proteus*, *Morganella*, and *Providencia* isolates, is very high. Although there are no specific data on the possible intrinsic resistance of these isolates to eravacycline, it is known that *Morganellaceae* exhibit intrinsic resistance to tetracyclines.

Considerable variability in resistance to the drug was observed among lactose non-fermenting Gram-negative bacterial isolates. This could be partially attributed to the fact that authors used different MIC resistance breakpoints, given the absence of published resistance breakpoints from the relevant organizations, resulting in non-uniform results. However, a meticulous evaluation of the data included in our tables reveals that the MIC_90_ values of eravacycline are rather low for *A. baumannii* complex isolates (i.e., not higher than 1 mg/L), including CRAB, in the majority of studies. The MIC_90_ values for eravacycline are also low for *Burkholderia cepacia* complex and *Stenotrophomonas maltophilia* isolates, although the published relevant information is limited. The data suggest that the MIC_90_ values for eravacycline in *P. aeruginosa* isolates are, as for other members or derivatives of the tetracycline class of antibiotics, higher than those for other lactose non-fermenting Gram-negative bacteria, especially *A. baumannii* isolates.

Previous studies have evaluated the use of tetracyclines and various tetracycline derivatives against *A. baumannii* [79]. It has been demonstrated that tetracyclines, often in combination with other antibiotics, exhibit promising effectiveness, particularly with minocycline and tigecycline [80,81]. In a systematic review of relevant published data, doxycycline or minocycline therapy was shown to be effective. In fact, it achieved clinical success in 77% of 156 patients with *A. baumannii* infections, including those involving the respiratory tract and bloodstream [82]. Additionally, TP-6076, another fluorocycline (like eravacycline), exhibited lower MIC values than tetracyclines and showed overall good antimicrobial activity against *A. baumannii* isolates. Specifically, the MIC_50_ and MIC_90_ values of TP-6076 for the studied 121 non-duplicate *A. baumannii* isolates were 0.03 mg/L and 0.06 mg/L, respectively [83].

Our evaluation of published data on resistance to eravacycline among Gram-negative bacterial isolates, including Enterobacterales and lactose non-fermenting isolates, suggests that in vitro antimicrobial susceptibility testing may help clinicians decide whether to initiate empirical therapy or continue targeted therapy with this drug. Additionally, the conduct of modern molecular microbiological diagnostic testing to detect resistance mechanisms can help clinicians make informed decisions.

### 4.2. Relevant Clinical Trial Data

Several Phase 3 clinical trials have been conducted to determine the effectiveness of eravacycline for the treatment of cIAIs and other infections. Three studies originate from China (ChiCTR2300078646, ChiCTR1900022060, ChiCTR2200055666) [84,85,86]. One clinical trial assessed the efficacy, safety, and tolerability of eravacycline versus ertapenem for the treatment of cIAI in hospitalized adults. Another study investigated the efficacy and safety of eravacycline for cIAI in ICU patients. A third study aimed to assess the efficacy, safety, and tolerability of eravacycline compared with moxifloxacin for the treatment of community-acquired bacterial pneumonia in adult patients. Neither of these studies have released results yet. Four other Phase 3 clinical trials were conducted by Tetraphase Pharmaceuticals, Inc. Two studies (NCT01844856, NCT02784704) evaluated the efficacy and safety of eravacycline in cIAIs compared to ertapenem and meropenem, respectively [87,88]. Two other studies (NCT01978938, NCT03032510) evaluated the efficacy and safety of eravacycline in complicated UTIs compared to levofloxacin and ertapenem, respectively [89,90]. The results of these studies highlighted its success in treating cIAIs relative to its comparators and its lack of success in treating cUTIs. As a result, it was approved for the sole indication of cIAIs.

An interventional, Phase 2 clinical study (NCT05537896) is currently underway to determine whether this antimicrobial agent can be used as a prophylactic treatment for patients with hematological malignancies who experience prolonged neutropenia [91]. As eravacycline has broad-spectrum activity but is not used for febrile neutropenia, it may be a good candidate for studies on prophylactic use in this patient population. Patients in this study will receive 1–1.5 mg/kg via 60 min intravenous infusion every 12 h. This treatment shall be continued until neutrophil recovery, febrile neutropenia, breakthrough infection, any grade 3–4 toxicity related to the medication, or completion of 21 days of therapy. As this study is still in the recruitment stage, no results have been published yet.

Concurrently, another Phase 2 clinical trial (NCT06794541) is evaluating the safety and tolerability of eravacycline in pediatric patients, specifically those aged 8 to 17 years with cIAI [92]. This Multicenter, Open-label trial has three patient cohorts. In one cohort (cohort 1), 1.5 mg/kg of the intravenous formulation of eravacycline will be administered as a single 60 min IV infusion to participants aged 12 to <18 years. In the second cohort (cohort 2a) and the third cohort (cohort 2b), 2 mg/kg will be administered in the same way for participants aged 10 to <12 years and 8 to <10 years, respectively. This study is currently in the recruitment stage, and therefore, no results are available yet.

### 4.3. Eravacycline Resistance Mechanisms

Eravacycline is not affected by classic tetracycline-specific efflux pumps including those encoded by the tet(A) and tet(B) genes. Also, its activity is not substantially affected by the ribosomal proteins such as those encoded by the tet(M) and tet(O) genes, that de-crease the binding of tetracyclines to the ribosomal target site. However, eravacycline resistance can occur by overexpression of other efflux pumps. *Acinetobacter* spp. and Enterobacterales produce the AdeABC and AcrAB-TolC pumps, respectively. Overexpression of such pumps can result in a rapid expulsion of the antibiotic from the bacterial cell [93]. Another resistance mechanism involves the enzymatic inactivation of eravacycline by the Tet(X) family of enzymes. These enzymes, especially Tet(X4), degrade eravacycline, rendering it ineffective. Bacteria can acquire genes encoding these enzymes via plasmids [94]. Resistance can also arise from other mutations which alter the target of eravacycline. For instance, mutations in 16S rRNA or other ribosomal proteins can alter the antibiotic’s binding site, thereby reducing its activity [95].

Additional research reveals that resistance mechanisms, often centered around mutations in key regulatory proteins, subsequently amplified the efflux activity, especially in carbapenem-resistant *K. pneumoniae* isolates. Specifically, in *K. pneumoniae*, resistance frequently develops due to mutations of the gene that encodes the Lon protease. These mutations reduce the functional expression or activity of the Lon protease, thereby increasing the level of the regulator RamA. This leads to the upregulation of the multidrug efflux system AcrA-AcrB-TolC and thereby reduces the intracellular accumulation of eravacycline. Additionally, eravacycline resistance in such isolates has also been associated with over-expression of OqxAB and MacAB efflux pumps. A frameshift mutation has also been identified in the DEAD/DEAH box helicase gene on a plasmid of an evolved resistant *K. pneumoniae* strain, which suggests that there can also be acquired resistance through altered ribosomal or RNA processing pathways [96]

### 4.4. Limitations

Our study is not without potential limitations. First, no universally accepted resistance breakpoints were available; therefore, many authors may have foregone reporting resistance percentages among the studied isolates and instead presented MIC ranges, MIC_50_, and MIC_90_. This casts some difficulty with the practical use of the evaluated data. However, the meticulous evaluation of published data on the resistance of various Gram-negative bacterial isolates to eravacycline that we present herein could help future researchers and clinicians appropriately use the antibiotic. Additionally, there is no universally accepted and validated risk-of-bias assessment tool for in vitro antimicrobial susceptibility studies; therefore, we did not use such a tool in our article. Finally, we had not registered the protocol of our study in a publicly available depository.

## 5. Conclusions

Eravacycline is a newer, fluorocycline-class antibiotic, approved for the treatment of patients with complicated intra-abdominal infections. The evaluation of the published evidence in our study suggests that this agent exhibits broad-spectrum antibacterial activity against most clinically important Gram-negative bacteria. It displayed high activity against *E. coli* isolates. However, notable levels of resistance were observed against *K. pneumoniae* and *E. cloacae* isolates. Lactose non-fermenting Gram-negative bacteria also had variable resistance against this drug. This could be attributed to the variability of the breakpoints used by the authors of the included studies, given the lack of established breakpoints of resistance. The aforementioned proportion of resistance, especially among selected Gram-negative bacterial isolates with advanced antimicrobial resistance patterns, suggests that in vitro antimicrobial susceptibility testing and modern molecular diagnostic tests for resistance mechanisms may aid optimal utilization of eravacycline in clinical practice.

## Figures and Tables

**Table 1:** Resistance of consecutive (non-selected) clinical isolates of Enterobacterales to eravacycline.

Author *	Year	Isolates	N	β-Lactamase Genes (*n* or %)	MIC Range/Value (mg/L)	MIC_50_ (mg/L)	MIC_90_ (mg/L)	Resistance [EUCAST] *n* (%)	Resistance [FDA] *n* (%)	Resistance [Author’s Criteria] ^a^ *n* (%)
Bianco [7]	2025	KPC-*K. pneumoniae*	264	KPC (264) ^b^	≤0.008–>0.5	0.5	>0.5	NA	NA	
Kinet-Poleur [8]	2025	Enterobacterales	222	NA;	≤0.06–>4	0.25	1	NA	ΝA	37 (16.7) proportion of strains inhibited by eravacycline at >0.5 mg/L.
		*K. pneumoniae*	95	OXA-48 (53), NDM (33), KPC (9), VIM (3);						
		*E. coli*	55	OXA-48 (33), NDM (17), VIM (2), KPC (1);						
		*C. freundii*	28	OXA-48 (22), VIM (6), NDM (3);						
		*E. cloacae* complex	25	VIM (9), OXA-48 (7), NDM (3);						
		*K. oxytoca*	10	OXA-48 (5), NDM (3), VIM (2), KPC (1);						
		Other ^c^	9	OXA-48 (7), NDM (1)						
Liao [9]	2024	*E. coli*	187	NA	0.015–8	0.25	0.5	18 (9.6)	18 (9.6)	
		*K. pneumoniae*	136		0.06–8	0.5	2	NA	57 (41.9)	
Chen [10]	2023	Enterobacterales	1202	CRE isolates: KPC + NDM (1), KPC-2 (43), NDM (20), IMP (3), OXA (2), no carbapenemase detected (32) ^d^	NA	0.125 0.25 0.25 NA	1 1 1 NA	NA	172 (14.3) 126 (21) 25 (21) NA	
		MDR isolates	599							
		CRE isolates	119							
		*Escherichia* spp.	284							
		*Klebsiella* spp.	243							
		*Salmonella* spp.	169							
		*Enterobacter* spp.	140							
		*Proteus* spp.	104							
		*Serratia* spp.	97							
		*Citrobacter* spp.	82							
		*Morganella* spp.	77							
		*Providencia* spp.	5							
		*Edwardsiella* spp.	1							
Zhanel [11]	2018	*E. coli*	1177	ESBL (141);	0.03–2	0.12	0.5	11 (0.9) NA	11 (0.9)	
		*K. pneumoniae*	381	ESBL (21);	0.06–8	0.25	0.5		38 (10)	
		*E. cloacae*	175	NA	0.06–8	0.5	1		32 (18.3)	
		*P. mirabilis*	91		0.5–4	1	2		89 (97.8)	
		*K. oxytoca*	88		0.06–1	0.25	0.5		2 (2.3)	
		*S. marcescens*	83		0.5–8	1	2		72 (86.7)	
		*E. aerogenes*	33		0.12–1	0.25	0.5		1 (3)	
		*M. morganii*	20		0.12–2	1	2		15 (75)	
		*C. freundii*	19		0.12–2	0.25	2		4 (21.1)	
Abdallah [12]	2015	*E. coli*	2866	KPC (5);	≤0.015–4	0.12	0.5	NA	NA	
		*K. pneumoniae*	944	KPC (124);	0.06–4	0.25	1			
		*E. cloacae*	124	KPC (4);	0.25–2	0.5	1			
		*E. aerogenes*	90	KPC (3)	0.12–2	0.25	1			
Solomkin [13]	2014	*E. coli*	86	NA	0.12–1	0.25	0.5	NA	NA	
		*K. pneumoniae*	14		0.25–2	0.5	1			
		*K. oxytoca*	6		0.25–1	0.5	1			
		*M. morganii*	3		1	1	1			
		*P. mirabilis*	2		0.5–1	NA	NA			
Sutcliffe [14]	2013	*E. coli*	445	CTX-M (53), TEM (35), OXA (16), SHV (22), CMY (13), NDM (2), ACT-5 (1), DHA-1 (1);	≤0.016–4	0.25	0.5	NA	NA	
		-								
		*K. pneumoniae*	394	CTX-M (29), TEM (17), OXA (6), SHV (57), KPC (20), NDM (3), DHA (1), FOX (1);	0.03–16	0.5	2			
		-								
		*E. cloacae*	270	NA	0.03–4	0.5	2			
		*P. mirabilis*	166		0.25–16	1	2			
		*C. freundii*	115		0.06–2	0.25	1			
		*S. marcescens*	112		0.25–8	1	1			
		*P. stuartii*	101		0.13–8	1	2			
		*E. aerogenes*	77		0.13–2	0.25	1			
		*P. vulgaris*	55		0.25–2	0.5	1			
		*K. oxytoca*	48		0.03–2	0.5	1			
		*M. morganii*	43		0.5–4	1	2			
		*Salmonella* spp.	30		0.13–0.5	0.25	0.25			
		*Shigella* spp.	30		0.06–1	0.13	0.5			

* Studies are presented in descending chronological order (and alphabetical order within a year). Abbreviations: ACT, *Enterobacter cloacae* AmpC type; *C. freundii*, *Citrobacter freundii*; CMY, *Citrobacter freundii* AmpC type; CRE, carbapenem-resistant Enterobacterales; CTX-M, cefotaximase-Munich; DHA, *Morganella darhamensis* AmpC β-lactamase; *E. aerogenes*, *Enterobacter aerogenes*; *E. cloacae* complex, *Enterobacter cloacae* complex; *E. coli*, *Escherichia coli*; ESBL, extended-spectrum β-lactamase; FOX, FOX-type AmpC β-lactamase; IMP, integron-mediated metallo-β-lactamase; *K. oxytoca*, *Klebsiella oxytoca*; *K. pneumoniae*, *Klebsiella pneumoniae*; KPC, *Klebsiella pneumoniae* carbapenemase; *M. morganii*, *Morganella morganii*; MDR, multidrug-resistant; MIC, minimum inhibitory concentration; NA, not available; NDM, New Delhi metallo-β-lactamase; OXA, oxacillinase; *P. mirabilis*, *Proteus mirabilis*; *P. stuartii*, *Providencia stuartii*; *P. vulgaris*, *Proteus vulgaris*; *S. marcescens*, *Serratia marcescens*; SHV, sulhydryl variable; spp., species; TEM, Temoniera β-lactamase; VIM, Verona integron-encoded metallo-β-lactamase. Notes: ^a^ According to the criteria, as defined by the authors in each study; ^b^ + SHV (25), TEM (21), NDM (1), OXA-48 (1), VIM (1) from KPC-Kp strains resistant to ceftazidime/avibactam and/or meropenem/vaborbactam and/or imipenem/relebactam; ^c^ *C. koseri* (3), *C. amalonaticus* (2), *K. aerogenes* (1), *K. variicola* (1), *R. ornitholytica* (1), *C. braakii* (1); ^d^ 88 carbapenemases produced by 87 isolates: 1 *K. pneumoniae* isolate produced two carbapenemases, KPC-2 and NDM-1.

**Table 2:** Resistance of selected clinical isolates of Enterobacterales to eravacycline.

Author *	Year	Isolates	N	β-Lactamase Genes (*n* or %)	MIC Range/Value (mg/L)	MIC_50_ (mg/L)	MIC_90_ (mg/L)	Resistance [EUCAST] *n* (%)	Resistance [FDA] *n* (%)	Resistance [Author’s Criteria] ^a^ *n* (%)
Ji [15]	2025	*E. coli*	500	NA	0.03–2	0.12	0.5	18 (3.6)	18 (3.6)	
		*K. pneumoniae*	500		0.03–16	0.5	2	NA	103 (20.6)	
Le Terrier [16]	2025	*E. coli*	110	CMY-like (94), NDM-5 (87), NDM-1 (20), NDM-7 (2), NDM-19 (1)	≤0.06–0.25	≤0.06	≤0.06	0 (0)	0 (0)	
Lee [17]	2025	*K. pneumoniae*	138	OXA-48-like + KPC (1), OXA-48-like + NDM (1), KPC (67), OXA-48-like (12), NDM (4), VIM (4);	0.125–8	1	2	NA	85 (61.6)	
		*E. coli*	29	KPC (2), NDM (2)	0.125–0.5	0.5	0.5		0 (0)	
García [18]	2024	CPE	399	OXA-48 (14), KPC-3 (4), VIM-1 (4) ^b^	≤0.12–8	1	2	NA	116 (29.1)	
		*K. pneumoniae*	293		0.12–8	1	2	NA	169 (57.7)	
		*E. cloacae complex*	54		0.12–8	1	4	NA	30 (55.6)	
		*E. coli*	18		0.12–1	0.25	0.5	1 (5.6)	1 (5.6)	
		*K. oxytoca*	14		0.12–2	0.5	2	NA	3 (23.1)	
		*C. freundii*	9		0.25–1	0.5	1		4 (44.4)	
		*S. marcescens*	7		0.5–2	1	2		3 (42.9)	
		*C. koseri*	2		0.5	0.5	0.5		0 (0)	
		*K. quasipneumoniae*	1		1	1	1		1 (100)	
		*K. variicola*	1		0.5	0.5	0.5		0 (0)	
Han [19]	2024	MDR *K. pneumoniae*	30	NA	0.25–32	2	16	NA	23 (76.7)	
Huang [29]	2024	CRKP	40	KPC (20), MBL (20) [including NDM (8), IMP (6), VIM (6)]	NA	2	4	15 (37.5)	15 (37.5)	
Markovska [21]	2024	*K. pneumoniae*	54	CTX-M-15 (43), NDM-1 (39), CMY-4 (24), KPC-2 (15), CTX-M-3 (6), CMY-99 (5), OXA-48 (5), VIM-1 (5)	≤0.25–1.5	0.38	1.5	NA	NA	59 (23.7) ≤0.5 mg/L for susceptibility and ≥1 mg/L for nonsusceptibility to eravacycline)
		*P. mirabilis*	5							
		*P. stuartii*	2							
		*C. freundii*	2							
		*E. cloacae * ^c^	1							
Markovska [22]	2024	*K. pneumoniae*	20	OXA-232 (16), NDM-5 (13), CTX-M-15 (5), KPC-2 (4)	NA	NA	NA	NA	17 (85)	
Słabisz [23]	2024	NDM-producing *K. pneumoniae*	60	NA	0.094–2	0.38	1	NA	16 (26.7)	0 (0) EUCAST (ECOFF) breakpoint of susceptibility was ≤2
Wu [24]	2024	*Enterobacterales*	5265	NA	NA	NA	NA	NA	NA	
		*CRE*	332	NA	0.032–8	0.125	0.25			
		*CRKP*	242	KPC (194), NDM (26), OXA (8), IMP (4), VIM (1);	NA	0.25	1			
		*CREC*	39	NDM (30);		NA	NA			
		*CR-others*	51	NDM (40), IMP (2), KPC (2), VIM (1)		0.125	0.5			
Wu [25]	2024	*K. pneumoniae*	59	KPC-2 (16), IMP-4 (7), IMP-8 (4), NDM-5 (2), IMP-26 (1), KPC-3 (1), KPC-4 (1), NDM-1 (10)	0.125–8	0.5	2	NA	23 (39)	
Zhang [26]	2024	CREC	31	NA	NA	NA	NA	NA	9 (29)	
		ESBL-producing *E. coli*	44						0 (0.0)	
		*E. cloacae*	16						7 (43.8)	
		*K. pneumoniae*	23						19 (82.6)	
		CRKP	22						22 (100)	
Bonnin [27]	2023	CR non-CPE Enterobacterales	284	CTX-M-15 (45), OXA-1 (43), TEM-1 (35), SHV-11 (21), SHV-1 (14), SHV-28 (10), DHA-1 (7), AmpC-like (4), AmpC (4), LAP-2 (3), OXY-2-16 (3), ACT-16 (2), ACT-45 (2), CMY-146 (2), CMY-2 (2), OXA-9 (2), OXY-2-19 (2), SHV-12 (2), SHV-187 (2), ACC-1A (1), ACC-1b-like (1), ACC-1c (1), ACT-24 (1), ACT-28 (1), ACT-56 (1), ACT-70 (1), ACT-C111 (1), ACT-C34 (1), ACT-C36-like (1), CMH-3 (1), CMY-42 (1), CTX-M-1 (1), CTX-M-14 (1), CTX-M-3 (1), CTX-M-33 (1), CTX-M-71 (1), CTX-M-8 (1), DHA-7 (1), LEN-43-like (1), MOX-9 (1), ORN-1 (1), OXA-10 (1), OXA-35 (1), OXA-392 (1), OXA-48 (1), OXA-9-like (1), SHV-2 (1), SHV-36 (1), synATM-fox (1), TEM-187-like (1), TEM_P3 (1), TEM PaPb (1)	≤0.25–8	0.5	4	NA	128 (45.1)	
		*K. pneumoniae*	145							
		*E. cloacae complex*	52							
		*E. coli*	32							
		*E. aerogenes*	28							
		*K. oxytoca*	11							
		*H. alvei*	10							
		*C. freundii* complex	4							
Hawser [28]	2023	Enterobacterales	12,436	NA	≤0.015–>16	0.25	0.5	808 (6.5)	808 (6.5)	
		*K. pneumoniae*	2040		0.06–>16	0.25	1	537 (26.3)	271 (13.3)	
		*E. coli*	2033		≤0.015–16	0.12	0.25	18 (0.9)	18 (0.9)	
		*K. oxytoca*	1948		0.03–16	0.25	0.25	72 (3.7)	72 (3.7)	
		*E. cloacae*	1881		0.03–8	0.25	1	198 (10.5)	198 (10.5)	
		*P. mirabilis*	1801		0.03–16	2	2	1670 (92.7)	1670 (92.7)	
		*K. aerogenes*	1786		0.03–8	0.25	0.5	114 (6.4)	114 (6.4)	
		*C. freundii*	1542		0.06–4	0.25	0.5	123 (8.0)	123 (8.0)	
		*C. koseri*	1206		0.06–2	0.25	0.25	8 (0.7)	8 (0.7)	
		*P. vulgaris*	375		0.25–4	1	1	252 (67.2)	252 (67.2)	
		*P. rettgeri*	322		0.25–16	2	2	303 (94.1)	303 (94.1)	
		*P. stuartii*	309		0.12–8	1	2	271 (87.7)	271 (87.7)	
		*S. marcescens*	214		0.5–16	1	2	168 (78.5)	168 (78.5)	
		*M. morganii*	209		0.12–4	0.5	2	93 (44.5) ^d^	93 (44.5)	
Huang [20]	2023	*Enterobacterales*	1000	NA;	0.125–16	0.5	1	323 (32.3)	323 (32.3)	
		*E. coli*	300	IMP (1), OXA-48 (1), VIM (1);	0.125–4	0.5	1	23 (7.7)	23 (7.7)	
		*K. pneumoniae*	300	KPC (22), OXA-48 (7), IMP (3), VIM (2);	0.125–16	0.5	4	143 (47.7)	143 (47.7)	
		*E. cloacae complex*	100	VIM (20), IMP (6), NDM (4), KPC (1);	0.25–8	0.5	3	56 (36)	56 (36)	
		*K. oxytoca*	100	NDM + IMP (1), IMP (9), NDM (3), KPC (2);	0.125–2	0.5	1	10 (10)	10 (10)	
		*C. freundii*	100	NA	0.125–4	0.25	1	11 (11)	11 (11)	
		*P. mirabilis*	100		2–16	4	4	0 (0) d	0 (0)	
Perez-Palacios [30]	2023	*K. pneumoniae*	43	OXA-1 (56), TEM-1 (52), CTX-M-15 (41), CMY-4 (21), OXA-48 (21), NDM-1 (12), SHV-28 (9), SHV-12 (8), SHV-11 (8), SHV-1 (8), ACT-25 (7), ACT-16 (4), NDM-7 (4), ACT-24 (3), SHV-119 (2), SHV-101 (2), SHV-27 (2), ACT-27 (2), ACT-45 (2), ACT-56 (2), ACT-70 (2), SHV-187 (1), SHV-76 (1), OXA-9 (1), ACT-36 (1), DHA-1 (1);	0.06–0.5	0.25	0.5	0 (0)	0 (0)	
		*E. hormaechei*	30	SHV-12 (3), TEM-1 (19), CTX-M-15 (15), OXA-1 (18), ACT-25 (7) ACT-24 (3) ACT-27 (2) ACT-45 (2) ACT-56 (2) ACT-70 (2) ACT-16 (2) ACT-36 (1), DHA-1 (1) CMY-4 (3), OXA-48 (9) NDM-1 (4) NDM-7 (4);	0.12–0.25	0.25	0.25	0 (0)	0 (0)	
		*E. coli*	3	NA	NA	NA	NA	NA	NA	
		*E. bugadensis*	1							
		*K. aerogenes*	1							
		*P. mirabilis*	1							
Zou [31]	2023	*K. pneumoniae*	160	KPC-2 (160);	≤0.0625–16	0.5	2	NA	75 (46.9)	
		*E. coli*	50	NDM-1 (47), KPC-2 (2), NDM-16 (1);	≤0.0625–4	0.25	0.5	4 (8)	4 (8)	
		*E. cloacae complex*	42	NDM-1 (33), other (5), KPC-2 (4)	0.25–8	1	4	NA	23 (54.8)	
Badran [32]	2022	Enterobacterales	156	NA	NA	NA	NA	NA	NA	
		NDM harboring	76						40 (52.6)	
		OXA-48 harboring	66						32 (48.5)	
		KPC harboring	12						10 (83.3)	
		IMP harboring	2						0 (0)	
Jurić [33]	2022	Total	80	OXA-48 (34), NDM (20), VIM (25), KPC (1);	≤0.5–8	≤0.5	4	NA	26 (32.5)	
		*K. pneumoniae*	43	OXA-48 (29), NDM (10), VIM (3), KPC (1);	≤0.5–8	≤0.5	4	NA	15 (34.9)	
		*E. cloacae*	12	VIM (9), NDM (3);	≤0.5–1	≤0.5	1	NA	2 (16.7)	
		*C. freundii*	12	VIM (8), NDM (4);	≤0.5–2	≤0.5	1	NA	5 (41.7)	
		*E. coli*	8	OXA-48 (5), NDM (2), VIM (1);	≤0.5–8	≤0.5	8	1 (12.5)	1 (12.5)	
		*K. oxytoca*	5	VIM (4), NDM (1)	≤0.5–4	1	4	NA	3 (60)	
Koreň [34]	2022	CR *K. pneumoniae*	41	CTX-M-15 (24), NDM-1 (24), OXA-1 (22), SHV-11 (22), TEM-1 (9), KPC-2 (8), SHV-12 (8), SHV-168 (4), TEM-156 (2), DHA-1 (1), KPC-3 (1), OXA-9 (1), TEM-116 (1)	0.25–2	0.5	0.5	NA	3 (7.3)	
Li [35]	2022	CR *E. coli*	20 ^e^	NDM−1 + NDM−7 (1), NDM−5 (14), NDM−1 (3), IMP−4 (1) (among the 19 clinical isolates);	0.12–4	0.25	1	3 (15)	3 (15)	
		CR *K. pneumoniae*	20 ^e^	KPC−2 (19) (among the 19 clinical isolates)	0.5–2	1	2	NA	NA	
Maraki [36]	2022	CRKP	266	NDM + VIM (3), KPC + NDM (2), KPC + VIM (2), KPC + OXA-48 (1), KPC (201), NDM (31), VIM (15), OXA-48 (11)	≤0.25–6	0.5	1.5	NA	90 (33.8)	
Johnston [37]	2021	Extended-spectrum cephalosporin-resistant *E. coli*	216	CTX-M group 1 + 9 (4), CTX-M group 1 (109), CTX-M group 9 (65)	0.03–>2	0.25	0.5	NA	6 (3)	
Kuo [38]	2021	*K. pneumoniae*	163	KPC-like (62), OXA48-like (19), IMP-like (5), VIM-like (5), NDM-like (1);	0.25–>8	1	2	NA	103 (63.2)	
		*E. coli*	17	IMP-like (5), VIM-like (5), OXA-48-like (2), NDM-like (1); ^f^	0.125–2	0.5	1		4 (23.5)	
Lee [39]	2021	*K. pneumoniae*	175	Among the total 87 CPE *K. pneumoniae* (83) and *E. coli* (4) isolates: KPC (69), OXA-48-like (12), NDM (4), VIM (4)	≤0.03–>64	16	>64	NA	NA	
		*E. coli*	26		1–>64	0.25	0.25	1 (3.8)	1 (3.8)	
Zalacain [40]	2021	Total	452	NDM (452)	≤0.06–16	0.5	2	NA	216 (47.7)	
		*K. pneumoniae*	275		0.125–16	0.5	2	NA	135 (49.1)	
		*E. coli*	59		0.125–2	0.25	1	8 (13.7)	8 (13.7)	
		*E. cloacae*	58		0.25–4	0.5	4	NA	26 (44.8)	
		Other ^g^	60		≤0.06–8	2	4		47 (78.3)	
Clark [41]	2020	Enterobacterales	122	KPC + MBL (7), KPC (70), MBL (20), other (25)	0.125–>8	1	4	NA	89 (73)	
		KPC	70		0.25–>8	1	4		53 (76)	
		MBL	20		0.25–4	1	4		13 (65)	
		Other resistance mechanism	25		0.25–>8	1	4		18 (72)	
		*Klebsiella* spp.	63		0.125–8	1	1		46 (73)	
		*Enterobacter* spp.	40		0.125–8	2	4		18 (72)	
		*Citrobacter* spp.	12		NA	NA	NA		NA	
		*Escherichia* spp.	6							
		*Serratia* spp.	1							
Johnston [42]	2020	CR *E. coli*	343	CTX-M (147), CMY-2 (96), KPC (54), OXA-48 (44), MBL (65) [including NDM (54), IMP (6), VIM (3)]	≤0.03–2	0.125	0.5	NA	8 (2)	
Morrisey [43]	2020	Enterobacterales	10,531	NA	0.03–16	0.25	0.5	779 (7.4)	779 (7.4)	
		*Klebsiella* spp.	4965		0.06–16	0.25	0.5	467 (9.4)	467 (9.4)	
		*E. coli*	1970		0.03–2	0.12	0.25	24 (1.2)	24 (1.2)	
		*Enterobacter* spp.	1820		0.06–8	0.5	1	189 (10.4)	189 (10.4)	
		*Citrobacter* spp.	1776		0.06–4	0.25	0.5	96 (5.4)	96 (5.4)	
		*P. mirabilis*	1348		0.12–>16	2	2	1205 (89.4)	1205 (89.4)	
		*S. marcescens*	948		0.12–8	1	2	850 (89.7) ^d^	850 (89.7)	
Zhao [44]	2019	*E. coli*	30	NA	NA	NA	NA	NA	NA	
		Carbapenem resistant	10		0.064–2	0.5	1			
		ESBL	10		0.064–0.25	0.125	0.25			
		Sensitive ^h^	10		0.064–0.25	0.064	0.125			
		*E. cloacae*	29		NA	NA	NA			
		Carbapenem resistant	1		0.5–0.5	0.5	0.5			
		ESBL	6		0.125–0.5	0.25	0.5			
		Sensitive ^h^	22		0.125–1	0.5	0.5			
		*K. pneumoniae*	49		NA	NA	NA			
		Sensitive ^h^	10		0.125–0.5	0.25	0.5			
		ESBL	10		0.125–2	0.5	1			
		KPC-2	9		0.25–4	0.5	2			
		NDM-1	3		0.5–1	0.5	1			
		mcr-1	4		0.5–16	1	16			
		Tigecycline resistant	13		2–16	8	16			
Johnson [45]	2016	*E. coli*	472	NA	NA	NA	NA	NA	NA	
		fluoroquinolone-resistant	238		0.03–1	0.25	0.5			
		fluoroquinolone-susceptible	234		0.03–0.5	0.13	0.25			
Livermore [46]	2016	*Klebsiella* spp.	120	KPC + ESBL (10), OXA-48 + ESBL (8), NDM + OXA-48 (1), VIM (20), NDM (19), OXA-48 (12), IMP (10), KPC (10);	0.06–2	0.25	1	NA	37 (30.8)	
		*Enterobacter* spp.	65	AmpC (10), KPC (10), NDM (10), OXA-48 (10), VIM (10), IMP (5);	0.06–0.5	0.13	0.25	NA	16 (24.6)	
		*E. coli*	60	ESBLs (10), KPC (10), NDM (10), OXA-48 (10), VIM (10);	0.06–1	0.25	0.5	1 (1.7)	1 (1.7)	
		*Proteaceae* spp.	15	NDM (8), OXA-48 (1), VIM (1);	0.06–2	0.25	1	NA	14 (93.3)	
		*Citrobacter* spp.	11	NDM (10), VIM (4), KPC (3), OXA-48 (2);	0.06–1	0.25	0.5	NA	7 (63.6)	
		*Serratia* spp.	9	KPC (2), NDM (2), OXA-48 (2)	0.06–0.5	0.13	0.5	NA	7 (77.8)	
Zhang [47]	2016	CRE	110	OXA + SHV + TEM + CTX-M-15 (2), OXA + SHV + TEM + AmpC (1), SHV + TEM + CTX-M-15 (4), OXA + SHV + TEM (2), SHV + TEM + AmpC (2), SHV + TEM + OXA (2), TEM + SHV + OXA (1), SHV + TEM (53), SHV + AmpC (3), TEM + AmpC (2), TEM + OXA (2), TEM + CTX-M-15 (2), SHV + CTX-M-15 (2), KPC-3 (91), TEM (24), KPC-2 (16), VIM-1 (8), SHV (4), SME-1 (3), NDM-1 (1), OXA (1)	0.5–4	1	2	NA	108 (98.2)	
		*K. pneumoniae*	96							
		*E. coli*	6							
		*S. marcescens*	6							
		*E. cloacae*	2							

* Studies are presented in descending chronological order (and alphabetical order within a year). Abbreviations: *C. freundii*, *Citrobacter freundiiρ*; *C. koseri*, *Citrobacter koseri*; CR, carbapenem resistant CRKP, carbapenem-resistant *Klebsiella pneumoniae*; CRE, Carbapenem-resistant Enterobacterales; CREC, Carbapenem-resistant *E. coli*; CTX-M, cefotaximase-Munich; CPE, carbapenemase-producing Enterobacterales; *E. aerogenes*, *Enterobacter aerogenes*; *E. bugadensis*, *Enterobacter bugadensis*; *E. cloacae*, *Enterobacter cloacae*; *E. cloacae complex*, *Enterobacter cloacae* complex; *E. coli*, *Escherichia coli*; *E. hormaechei*, *Enterobacter hormaechei*; ESBL, extended-spectrum β-Lactamase; *H. alvei*, *Hafnia alvei*; IMP, integron-mediated metallo-β-lactamase; *K. aerogenes*, *Klebsiella aerogenes*; *K. oxytoca*, *Klebsiella oxytoca*; *K. pneumoniae*, *Klebsiella pneumoniae*; *K. quasipneumoniae*, *Klebsiella quasipneumoniae*; *K. variicola*, *Klebsiella variicola*; KPC, *Klebsiella pneumoniae* carbapenemase; LAP, lectoferrin antibody positive; LEN, local *Enterobacteriaceae*; *M. morganii*, *Morganella morganii*; MBL, Metallo-β-Lactamase; mcr-1, mobilized colistin resistance gene, type 1; MDR, multidrug-resistant; MIC, minimum inhibitory concentration; MOX, *Morganella moxii*-related AmpC β-lactamase; NA, not available; NDM, New Delhi metallo-β-lactamase; ORN, *Raoultella ornithinolytica* β-lactamase; OXA, oxacillinase; OXA-like, oxacillinase-like; OXY, *Klebsiella oxytoca* β-lactamase; *P. mirabilis*, *Proteus mirabilis*; *P. rettgeri*, *Providencia rettgeri*; *P. stuartii*, *Providencia stuartii*; *P. vulgaris*, *Proteus vulgaris*; *S. marcescens*, *Serratia marcescens*; SHV, sulhydryl variable; SME, *Serratia marcescens* β-lactamase; spp., species; SYNATM-fox, FOX-type AmpC β-lactamase; TEM, Temoniera β-lactamase; VIM, Verona integron-encoded metallo-β-lactamase. Notes: ^a^ According to the criteria, as defined by the authors in each study; ^b^ from the 24 *Enterobacterales* isolates chosen for in-depth study because tigecycline and/or eravacycline showed low activity against them; ^c^ Three *P. mirabilis* and two *P. stuartii* isolates were excluded from this testing, meaning 59 isolates were tested; ^d^ used the *E. coli* EUCAST breakpoints of resistance MIC > 0.5 mg/L for all Enterobacterales; ^e^ 19 clinical isolates and 1 carbapenemase-producing strain; ^f^ for both *E. coli* and *K. pneumoniae:* AmpC +/ESBL (85), ESBL + AmpC (43), AmpC (41), ESBL (1); ^g^ *C. freundii* (5), *E. asburiae* (1), *Enterobacter* spp. (2), *K. aerogenes* (4), *K. oxytoca* (9), *M. morganii* (4), *P. mirabilis* (6), *P. rettgeri* (4), *P. stuartii* (15), *S. marcescens* (10); ^h^ do not have ESBL and carbapenem resistance.

**Table 3:** Resistance of consecutive (non-selected) clinical isolates of lactose non-fermenting Gram-negative bacteria to eravacycline.

Author *	Year	Isolates	N	β-Lactamase Genes (*n* or %)	MIC Range/Value (mg/L)	MIC_50_ (mg/L)	MIC_90_ (mg/L)	Resistance [Authors’ Criteria] ^a^ *n* (%)
Ataman [48]	2025	*A. baumannii*	100	NA	0.25–32	4	8	NA
Buyukyanbolu [49]	2025	*A. baumannii* complex	523	NA	NA	0.5	1	NA
Ji [15]	2025	*A. baumannii*	500	NA	0.004–4	0.06	0.5	NA
Kinet-Poleur [8]	2025	*Total*	53	CP-CRAB (29) [including OXA-23 (15), OXA-24 (6), NDM (6), OXA-58 (2)], non-CP CRAB (4);	<0.06–2	0.25	1	23 (43.4) ^b^ 14 (28.3) ^c^
		*A. baumannii*	50	As above	NA	NA	NA	NA
		Other *Acinetobacter* spp. ^d^	3	None	NA	NA	NA	NA
Li [50]	2025	*B. cenocepacia*	102	NA	0.25–4	0.5	1	NA
		*B. multivorans*	95		0.25–4	0.5	1	
		*B. contaminans*	27		0.25–2	0.5	2	
Mataracı-Kara [51]	2025	*P. aeruginosa*	40	VIM (12), OXA 23-58 (8), OXA 198-10-427 (7), NDM (4), PER (3), SHV (3), OXA 51 (2), GES (1), IMP (1), OXA 48 (1)	8–128	32	64	NA
Gautam [52]	2024	*A. baumannii*	48	NA	0.125–4	0.25	2	19 (38.6) ^e^
Liao [9]	2024	*A. baumannii*	58	NA	0.03–4	0.25	2	NA
Tsai [53]	2024	*S. maltophilia*	52	NA	<0.03–4	0.5	2	NA
Tunney [54]	2024	*Burkholderia* spp.	106	NA	NA	0.5	>0.5	NA
		*B. multivorans*	49			NA	NA	
		*B. cenocepacia*	28					
		*B. cepacia*	15					
		*B. gladioli*	3					
		*B. vietnamiensis*	10					
		Other	1					
		*Stenotrophomonas* spp.	102			0.25	>0.5	
		*S. maltophilia*	102			0.25	>0.5	
		*Achromobacter* spp.	74			0.5	>0.5	
		*A. xylosoxidans*	69			NA	NA	
		Other	5					
		*Pandoraea* spp.	11					
		*P. apista*	2					
		*P. pnomenusa*	1					
		*P. pulmonicola*	5					
		*P. sputorum*	3					
		*Ralstonia* spp.	7					
		*R. mannitolilytica*	6					
		*R. picketti*	1					
Zalacain [55]	2024	*A. baumannii*	500	NA	≤0.125–4	1	2	NA
		CRAB	363		≤0.125–4	1	2	
Galani [56]	2023	*A. baumannii*	271	OXA-51 (271), OXA-23 (268), TEM (162), NDM (4)	0.06–>32	2	4	NA
Hawser [28]	2023	*A. baumannii*	1893	NA	≤0.015–8	0.5	1	NA
		*S. maltophilia*	356		0.06–8	0.5	2	
Deolankar [57]	2022	*A. baumannii*	19	NA	NA	0.9	3	NA
		CRAB	7		NA	NA	NA	NA
Lee [58]	2020	CR *A. nosocomialis*	89	NA	NA	NA	NA	NA
		ST410	61		0.06–8	0.13	0.25	
		ST1272	15		0.03–1	0.06	0.25	
		Other types ^f^	13		0.03–2	0.13	0.5	
Morissey [43]	2020	*A. baumannii*	2097	NA	≤0.015–16	0.5	1	NA
		*P. aeruginosa*	1647		0.015–16	8	16	
		*S. maltophilia*	1210		0.03–16	1	1	
Zhanel [11]	2018	*S. maltophilia*	118	NA	0.25–16	1	4	NA
		*A. baumannii*	28		0.03–1	0.06	0.5	
Livermore [46]	2016	CRAB	55	OXA-23/40/51/58 (39), NDM (5), OXA-23 (5)	0.06–2	0.5	1	NA
Abdallah [12]	2014	*A. baumannii*	158	OXA-23-like (58), OXA-24-like (2), KPC (1)	≤0.015–8	0.5	1	NA
Solomkin [13]	2014	*A. baumannii* complex	4	NA	0.25–0.5	0.5	0.5	NA
		*P. aeruginosa*	6		4–16	16	NA	
		*C. testosteroni*	2		0.015–0.03	16	NA	
Sutcliffe [14]	2013	*A. baumannii*	188	NA	0.016–8	0.25	1	NA
		*P. aeruginosa*	145		1–>32	8	32	
		*S. maltophilia*	105		≤0.016–8	0.5	2	
		*A. lwoffii*	34		0.03–0.25	0.13	0.25	
		*B. cenocepacia*	10		0.13–32	8	32	

* Studies are presented in descending chronological order (and alphabetical order within a year). Abbreviations: *A. baumannii*, *Acinetobacter baumannii*; *A. lwoffii*, *Acinetobacter lwoffii*; *A. xylosoxidans*, *Achromobacter xylosoxidans*; *B. cenocepacia*, *Burkholderia cenocepacia*; *B. contaminans*, *Burkholderia contaminans*; *B. cepacia*, *Burkholderia cepacia*; *B. gladioli*, *Burkholderia gladioli*; *B. multivorans*, *Burkholderia multivorans*; *B. vietnamiensis*, *Burkholderia vietnamiensis*; *C. testosteroni*, *Comamonas testosteroni*; CRAB, Carbapenem-Resistant *Acinetobacter baumannii*; GES, Guiana extended-spectrum β-lactamase; IMP, imipenemase; KPC, *Klebsiella pneumoniae* carbapenemase; MIC, minimum inhibitory concentration; NA, not available; NDM, New Delhi metallo-β-lactamase; non-CP, non-carbapenemase-producing; OXA, oxacillinase; *P. aeruginosa*, *Pseudomonas aeruginosa*; *P. apista*, *Pandoraea apista*; *P. pnomenusa*, *Pandoraea pnomenusa*; *P. pulmonicola*, *Pandoraea pulmonicola*; *P. sputorum*, *Pandoraea sputorum*; PER, *Pseudomonas* extended-spectrum β-lactamase; *R. mannitolilytica*, *Ralstonia mannitolilytica*; *R. pickettii*, *Ralstonia pickettii*; SHV, sulfhydryl variable β-lactamase; VIM, Verona integron-encoded metallo-β-lactamase. Notes: ^a^ According to the criteria, as defined by the authors in each study; ^b^ if S ≤ 0.25 mg/L [Tentative ECOFF determined by Jing R. et al. [76]]; ^c^ if S ≤ 0.5 mg/L [PK/PD breakpoints according to CASFM]; ^d^ 2 *Acinetobacter pittii*, 1 *Acinetobacter junii*; ^e^ the authors used the Enterobacterales EUCAST breakpoints; ^f^ ST433 (6) ST68 (5) and ST217 (2).

**Table 4:** Resistance of selected clinical isolates of lactose non-fermenting Gram-negative bacteria to eravacycline.

Author *	Year	Isolates	N	β-Lactamase Genes (*n* or %)	MIC Range/Value (mg/L)	MIC_50_ (mg/L)	MIC_90_ (mg/L)	Resistance [Authors’ Criteria] ^a^ *n* (%)
Liu [59]	2025	CRAB	587	NA	0.06–2	0.5	1	11 (1.9) ^b^
Yin [60]	2025	CRAB	48	OXA-51 (48), OXA-23 (43)	≤0.0625–4	0.5	1	NA
Chen [61]	2024	*A. baumannii*	492	OXA-51 (9), OXA−23 (5), OXA-24 (4), NDM-1 (1), OXA-58 (1), VIM (1)	NA	NA	NA	NA
		CRAB	253	OXA-23 (6), OXA-51 (6), OXA-24 (3), NDM-1 (1), OXA-58 (1), VIM (1)	0.12–16	0.5	1	6 (2.4) ^c^
		CSAB	239	OXA-51 (3), OXA-24 (1)	0.03–8	0.12	0.5	3 (1.3)
García [18]	2024	*A. baumannii*	118	OXA-201 (2), OXA-58 (2) ^d^	≤0.25–4	≤0.25	1	NA
Halim [62]	2024	CRAB	21	OXA-66 (6), ADC-73 (4), OXA-23 (4), ADC-30 (1), ADC-33 (1), ADC-80 (1), ADC-150 (1), OXA-94 (1), OXA-421 (1) ^e^	0.75–32	5	16	11 (52.4) ^f^
Li [63]	2024	*A. baumannii*	287	OXA-23 (25), OXA-24 (13), OXA-48 (2), OXA-58 (2), IMP (1), KPC (1) ^g^	NA	NA	NA	NA
		CRAB	147		0.06–16	0.25	1	3 (2), I 2 (1.4)
		CSAB	140		0.01–8	0.12	0.5	1 (0.7), I 2 (1.4) ^h^
Sun [64]	2024	*A. baumannii*	45	ADC (45), OXA-51 (45), OXA-23 (39), OXA-23 + OXA- 58 (1), NDM-1 (1), OXA-24 (1)–CRAB (42)	<0.03–2	0.12	1	4 (8.9) ^i^
Wu [24]	2024	*A. baumannii*	699	NA	NA	NA	NA	NA
		CRAB	440	OXA-23 (440), NDM (11)	0.032–8	0.25	1	
Zhang [26]	2024	CRAB	29	NA	NA	NA	NA	7 (24.1) ^j^
Camargo	2023	*P. aeruginosa*	119	OXA (119), AmpC (119), CTX-M-2 (10), KPC-2 (2), GES-1 (1)	0.5–32	>64	>64	NA
Chandran [66]	2023	CRAB	150	OXA-23 like + NDM (77), OXA-23 like (66), OXA 58-like + NDM (3), OXA-51 (150), VIM (4),	≤0.03–1	0.25	0.5	NA
Chew [67]	2023	*P. aeruginosa*	34	IMP-1 (10), NDM (8);	8–>8	>8	>8	NA
		*A. baumannii*	28	OXA-23 + NDM (2), OXA-58 + NDM (1), OXA-23 (3), IMP (1)	0.03–>8	0.5	2	
		*S. maltophilia*	15	NA	0.06–8	0.5	8	
		*E. anophelis*	7		0.5–2	2	2	
Gopikrishnan [68]	2023	CRAB	52	NA	NA	NA	NA	NA
		OXA-23 producers	44		0.015–0.5	0.25	4	
		OXA-58-like producers	4		0.03–0.25	1	2	
		OXA-23 + OXA-58-like producers	3		0.5 ^k^	0.5	0.5	
		OXA-24 producers	1		0.03	0.03	0.03	
Wu [69]	2023	*S. maltophilia*	77	NA	0.03–16	2	4	NA
Li [35]	2022	CRAB	20	OXA-51 (20), OXA-23 (17)	0.5–2	1	1	NA
Liu [70]	2022	*A. baumannii*	255	NA	≤0.03–4	0.5	1	NA
		*P. aeruginosa*	150		0.5–32	8	16	NA
Yin [71]	2022	*A. baumannii* complex	13	NA	2–8	4	8	NA
Kuo [38]	2021	*A. baumannii* (imipenem-non-susceptible)	136	OXA-23-like (117), OXA-24-like (22), OXA-51-like (11), OXA-59-like (1)	0.125–8	1	2	110 (80.9) ^l^
Biagi [72]	2020	*S. maltophilia*	41	NA	0.5–16	2	8	NA
Seifert [73]	2020	CRAB	323	OXA-23 (256), OXA-58 (33), OXA-40 (23), OXA-51 (7; overexpressed), NDM (3)	0.03–8	0.5	1	NA
Zhao [44]	2019	*A. baumannii*	39	NA	NA	NA	NA	NA
		Sensitive to carbapenems and tigecycline	9		0.016–0.25	0.125	0.25	
		OXA-23 positive	21		0.5–2	1	2	
		Tigecycline resistant	9		2–4	2	2	
Seifert [74]	2018	CRAB	286	OXA-23 (231), OXA-58 (27), OXA-40 (17), OXA-51 (9; overexpressed)	≤0.06–8	0.5	1	NA

* Studies are presented in descending chronological order (and alphabetical order within a year). Abbreviations: *A. baumannii*, *Acinetobacter baumannii*; ADC, *Acinetobacter* derived cephalosporinase; CRAB, carbapenem-resistant *Acinetobacter baumannii*; CSAB, carbapenem-susceptible *Acinetobacter baumannii*; *E. anophelis*, *Elizabethkingia anophelis*; I, intermediate resistance; IMP, imipenemase; KPC, *Klebsiella pneumoniae* carbapenemase; MIC, minimum inhibitory concentration; NA, not available; OXA, oxacillinase; *P. aeruginosa*, *Pseudomonas aeruginosa*; *S. maltophilia*, *Stenotrophomonas maltophilia*. Notes: ^a^ According to the criteria, as defined by the authors in each study; ^b^ ChinaCAST breakpoints; ^c^ S ≤ 2 mg/L, I = 4 mg/L, R ≥ 8 mg/L; ^d^ Regarding the four *A. baumannii* isolates selected explicitly for in-depth study due to low activity against tigecycline and/or eravacycline; ^e^ Regarding the selected eight strains for whole genome sequencing analysis; ^f^ MIC value of ≤4.0 mg/L as a proxy to consider strains “susceptible” for eravacycline (this proxy was established based on the CLSI cutoffs for other drugs within the tetracycline class); ^g^ for a subset of 25 eravacycline heteroresistant CRAB isolates; ^h^ As *Acinetobacter baumannii* MIC breakpoints for eravacycline and tigecycline have not yet been established by CLSI and FDA, this study categorized the MIC values into three levels based on reported breakpoints (Marchaim et al., 2014 [78]; Abdallah et al., 2015 [12]): ≤2 mg/L (sensitive, S), 4 mg/L (intermediate, I), and ≥8 mg/L (resistant, R); ^i^ based on Enterobacterales EUCAST breakpoints; ^j^ the thresholds from a reference study (Zhanel et al., 2018 [11]) were adopted: ERV susceptible at ≤0.5 mg/L and ERV resistant at >0.5 mg/L; ^k^ All three isolates had MIC of 0.5 mg/L; ^l^ FDA breakpoints for Enterobacterales were used.

## Data Availability

No new data were created or analyzed in this study.

## Supplementary Materials

The following supporting information can be downloaded at: https://www.mdpi.com/article/10.3390/pathogens14121214/s1, Supplementary File S1: Detailed search strategies used in each resource as of 29 August 2025; Supplementary File S2: PRISMA 2020 abstract checklist [75]. Supplementary File S3: PRISMA 2020 checklist [75].

## Abbreviations

*A. baumannii*	*Acinetobacter baumannii*
cIAI	complicated intra-abdominal infections
CLSI	Clinical and Laboratory Standards Institute
CRAB	carbapenem-resistant *A. baumannii* isolates
CSAB	carbapenem-susceptible *A. baumannii*
DOIs	digital object identifiers
*E. cloacae*	*Enterobacter cloacae*
*E. coli*	*Escherichia coli*
EMA	European Medicines Agency
EUCAST	European Committee on Antimicrobial Susceptibility Testing
FDA	Food and Drug Administration
*K. pneumoniae*	*Klebsiella pneumoniae*
MIC	minimum inhibitory concentration
*P. aeruginosa*	*Pseudomonas aeruginosa*
