# Supplementary material for: Resistance of Gram-Negative Bacteria to Eravacycline: A Systematic Review of Data from In Vitro Studies
Source: Pathogens. 2025 Nov 28;14(12):1214. doi: 10.3390/pathogens14121214 (PMC12735846; doi:10.3390/pathogens14121214)
Supplement: Supplementary file 1 [file pathogens-14-01214-s001.zip › Supplementary File S1.pdf]

Supplementary File S1. Detailed search strategies used in each resource as of 29 August 2025

Resource	Search string	Coverage (years)	Results
Embase	('eravacycline'/exp OR eravacycline OR 'tp 434'/exp OR 'tp 434') AND ('antibiotic resistance'/exp OR 'antibiotic resistance' OR 'resistance'/exp OR resistance OR 'non-susceptibility' OR 'non susceptibility' OR nonsusceptibility OR 'reduced susceptibility') AND ('enterobacteriaceae'/exp OR enterobacteriaceae OR 'enterobacterales'/exp OR enterobacterales OR 'gram-negative' OR 'multidrug-resistant' OR 'pseudomonas'/exp OR pseudomonas OR 'acinetobacter'/exp OR acinetobacter OR 'stenotrophomonas'/exp OR stenotrophomonas OR 'lactose non fermenting' OR 'lactose non-fermenting' OR 'lactose-non-fermenting') AND ('mic'/exp OR mic OR 'minimum inhibitory concentration'/exp OR 'minimum inhibitory concentration' OR 'disc diffusion'/exp OR 'disc diffusion')	1947 –present	259
PubMed	(eravacycline OR TP-434) AND ("antibiotic resistance" OR resistance OR "non-susceptibility" OR "non susceptibility" OR nonsusceptibility OR "reduced susceptibility") AND (Enterobacteriaceae OR Enterobacterales OR "Gram-negative" OR "multidrug-resistant" OR Pseudomonas OR Acinetobacter OR Stenotrophomonas OR "lactose non fermenting" OR "lactose non-fermenting" OR "lactose-non-fermenting") AND (MIC OR "minimum inhibitory concentration" OR "disc diffusion")	1946 –present	59
Scopus	(eravacycline OR TP-434) AND ("antibiotic resistance" OR resistance OR "non-susceptibility" OR "non susceptibility" OR nonsusceptibility OR "reduced susceptibility") AND (Enterobacteriaceae OR Enterobacterales OR "Gram-negative" OR "multidrug-resistant" OR Pseudomonas OR Acinetobacter OR	1966 –present	197

	Stenotrophomonas OR "lactose non fermenting" OR "lactose non-fermenting" OR "lactose-non-fermenting") AND (MIC OR "minimum inhibitory concentration" OR "disc diffusion")		
Web of Science	(eravacycline OR TP-434) AND ("antibiotic resistance" OR resistance OR "non-susceptibility" OR "non susceptibility" OR nonsusceptibility OR "reduced susceptibility") AND (Enterobacteriaceae OR Enterobacterales OR "Gram-negative" OR "multidrug-resistant" OR Pseudomonas OR Acinetobacter OR Stenotrophomonas OR "lactose non fermenting" OR "lactose non-fermenting" OR "lactose-non-fermenting") AND (MIC OR "minimum inhibitory concentration" OR "disc diffusion")	1900 – present	35
